# Supplementary material for: Identification of photoperiod-induced specific miRNAs in the adrenal glands of Sunite sheep (Ovis aries)
Source: Front Vet Sci. 2022 Jul 22;9:888207. doi: 10.3389/fvets.2022.888207 (PMC9354845; doi:10.3389/fvets.2022.888207)
Supplement: Supplementary file 1 [file Data_Sheet_1.ZIP › Supplementary file/miRDeep2 file.zip!/miRDeep2 file/Novel_Structure/LP42A3/Novel_81.pdf]

Provisional ID	:	NC_019484.2_24876
Score total	:	80
Score for star read(s)	:	3.9
Score for read counts	:	73
Score for mfe	:	1.5
Score for randfold	:	1.6
Score for cons. seed	:	
Total read count	:	155
Mature read count	:	132
Loop read count	:	0
Star read count	:	23

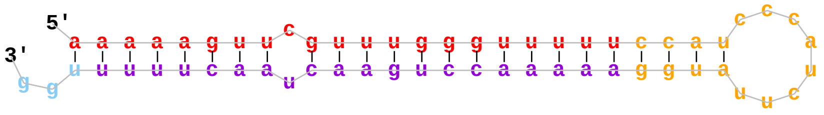

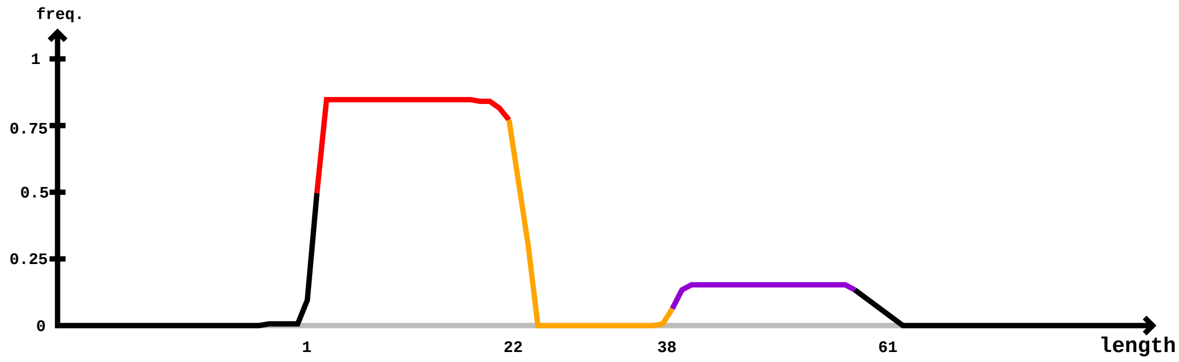

Mature **Star**

```
5'- uaaaaaauaaaaauauggauuggacaaaaaguucguuuggguuuuuccaucccaucuuauggaaaaaccugaacuaacuuuuuggucaacccaguauauacugucuccaaga  -3'  obs
    uaaaaaauaaaaauauggauuggacaaaaaguucguuuggguuuuuccaucccaucuuauggaaaaaccugaacuaacuuuuuggucaacccaguauauacugucuccaaga       exp
    ...............((.(((..(((((((((.((((((((((((((((........)))))))))))))))).)))))))))..)))))((((....))))..........  reads  mm  sample
    ....................uggacaaaaaguucguuugggu......................................................................  1      0   seq
    ........................caaaaaguucguuuggguuu....................................................................  1      0   seq
    ........................caaaaaguucguuuggguuuu...................................................................  4      0   seq
    ........................caaaaaguucguuCggguuuuu..................................................................  1      1   seq
    ........................caaaaaguucguuuggguuuuu..................................................................  8      0   seq
    .........................aaaaaguucguuuggguuu....................................................................  3      0   seq
    .........................aaaaaguucguuCggguuuu...................................................................  1      1   seq
    .........................aaaaaguucguuuggguuuuu..................................................................  52     0   seq
    .........................aaaaaguucguuugUguuuuu..................................................................  1      1   seq
    .........................aaaaaguucguuuggguuuCuc.................................................................  1      1   seq
    .........................aaaaaguucguuuggguuCuuc.................................................................  1      1   seq
    .........................aaaaaguucguuuggguuuuucA................................................................  4      1   seq
    ..........................aaaaguucguuuggguuuC...................................................................  2      1   seq
    ..........................aaaaguucguuuggguuuuuU.................................................................  10     1   seq
    ..........................aaaaguucguuuggguuuuucA................................................................  41     1   seq
    ..........................aaaaguucguuuggguuuuucG................................................................  2      1   seq
    .............................................................gaaaaaccugaacGaacuuuu..............................  1      1   seq
    ..............................................................aaaaaccugaacGaacuuu...............................  1      1   seq
    ..............................................................aaaaaccugaacAaacuuuu..............................  1      1   seq
    ..............................................................aaaaaccugaacGaacuuuu..............................  7      1   seq
    ...............................................................aaaaccugaacAaacuuu...............................  2      1   seq
    ...............................................................aaaaccugaacAaacuuuu..............................  1      1   seq
    ...............................................................aaaaccugaacuaacuuuuC.............................  1      1   seq
    ...............................................................aaaaccugaacGaacuuuuugg...........................  1      1   seq
    ...............................................................aaaaccugaacuaacuuuuugA...........................  6      1   seq
    ................................................................aaaccugaacAaacuuuuuggu..........................  3      1   seq
```
